# Supplementary material for: A mathematical perspective on edge-centric brain functional connectivity
Source: Nat Commun. 2022 May 16;13:2693. doi: 10.1038/s41467-022-29775-7 (PMC9110367; doi:10.1038/s41467-022-29775-7)
Supplement: Supplementary file 1 — Supplementary Information [file 41467_2022_29775_MOESM1_ESM.pdf]

SUPPLEMENTARY MATERIAL

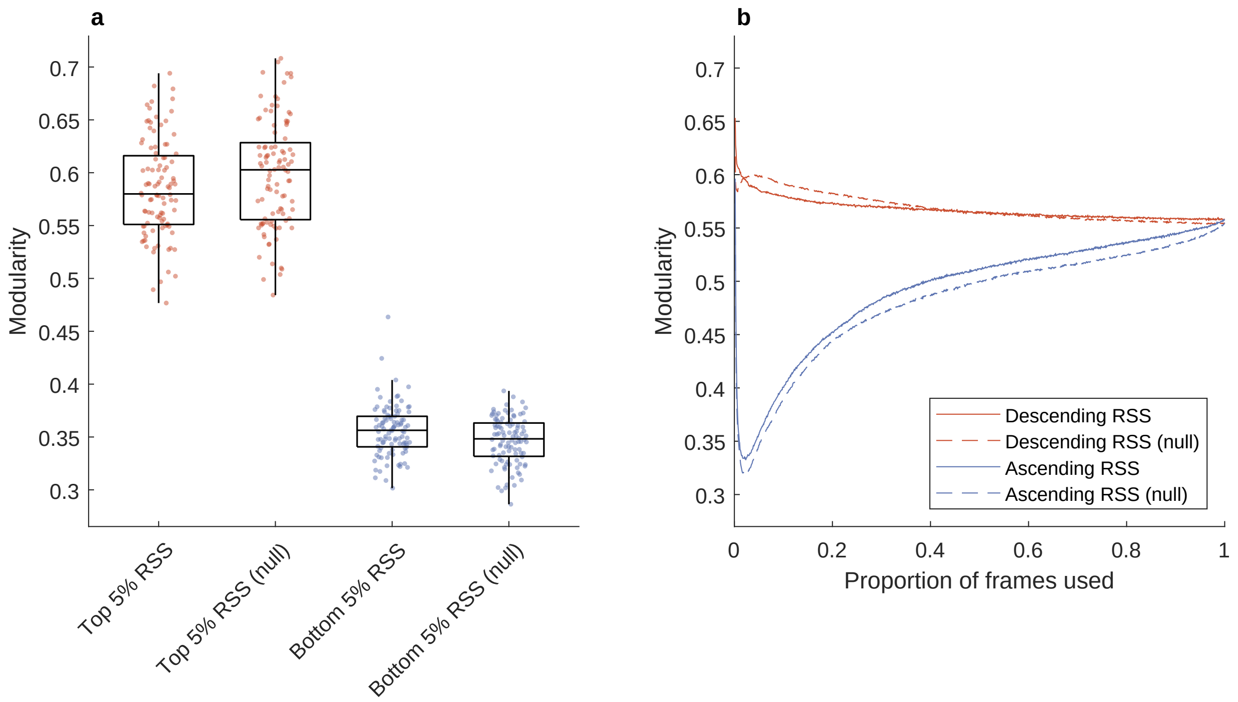

Supplementary Fig. 1. The static Gaussian null model can reproduce the empirical network modularity, which can be interpreted as a measure of the segregation between the network systems. The employed q^* variant of modularity is well suited for use with correlation matrices [49]. **a)** The empirical results match the finding published in [15, Fig. 1E]: the networks estimated using the top 5% of frames exhibit much higher modularity than those estimated using the bottom 5% of frames. The gap is accurately replicated by the null model. Each point corresponds to one of 100 unrelated subjects from the Human Connectome Project (HCP) dataset, with boxes indicating the quartiles and whiskers length specified as 1.5 times the interquartile range. **b)** The same results hold more generally when the frames are ordered according to the corresponding RSS amplitude, either in descending or ascending order. Here, the curves represent the average over 100 subjects.

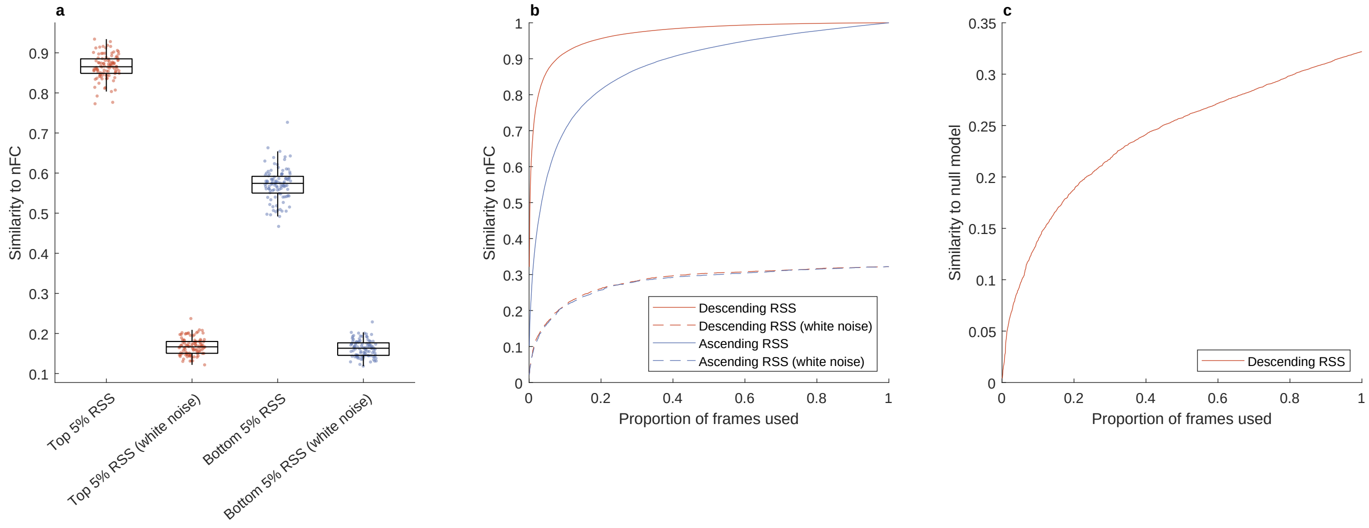

Supplementary Fig. 2. Accounting for spatial correlations is essential to reproduce the empirical edge-centric findings. To prove that, we repeated the analysis reported in Fig. 3 but ignored the spatial correlations in addition to the temporal ones. As a result, the differences between frames exhibiting high and low cofluctuation magnitudes (RSS) vanished. **a)** When using spatially uncorrelated white noise, the small fraction of frames exhibiting the largest RSS is no longer able explain most of the nFC variance (unlike the null model presented in the main text, which accounts for the observed spatial correlations encoded in the nFC matrix). The similarity is computed as the Pearson correlation coefficient between the nFC and the average FC estimated from the top and bottom 5% of the total frames. Each point corresponds to one of 100 unrelated subjects from the Human Connectome Project (HCP) dataset, with boxes indicating the quartiles and whiskers length specified as 1.5 times the interquartile range. **b)** The same inability is shown more generally when the frames are ordered according to the corresponding RSS amplitude, either in descending or ascending order. Here, the curves represent the average similarity over 100 subjects. **c)** Unlike the spatially-correlated null model, the top frames generated by Gaussian white noise do not exhibit high similarity to the top frames of the real HCP data.

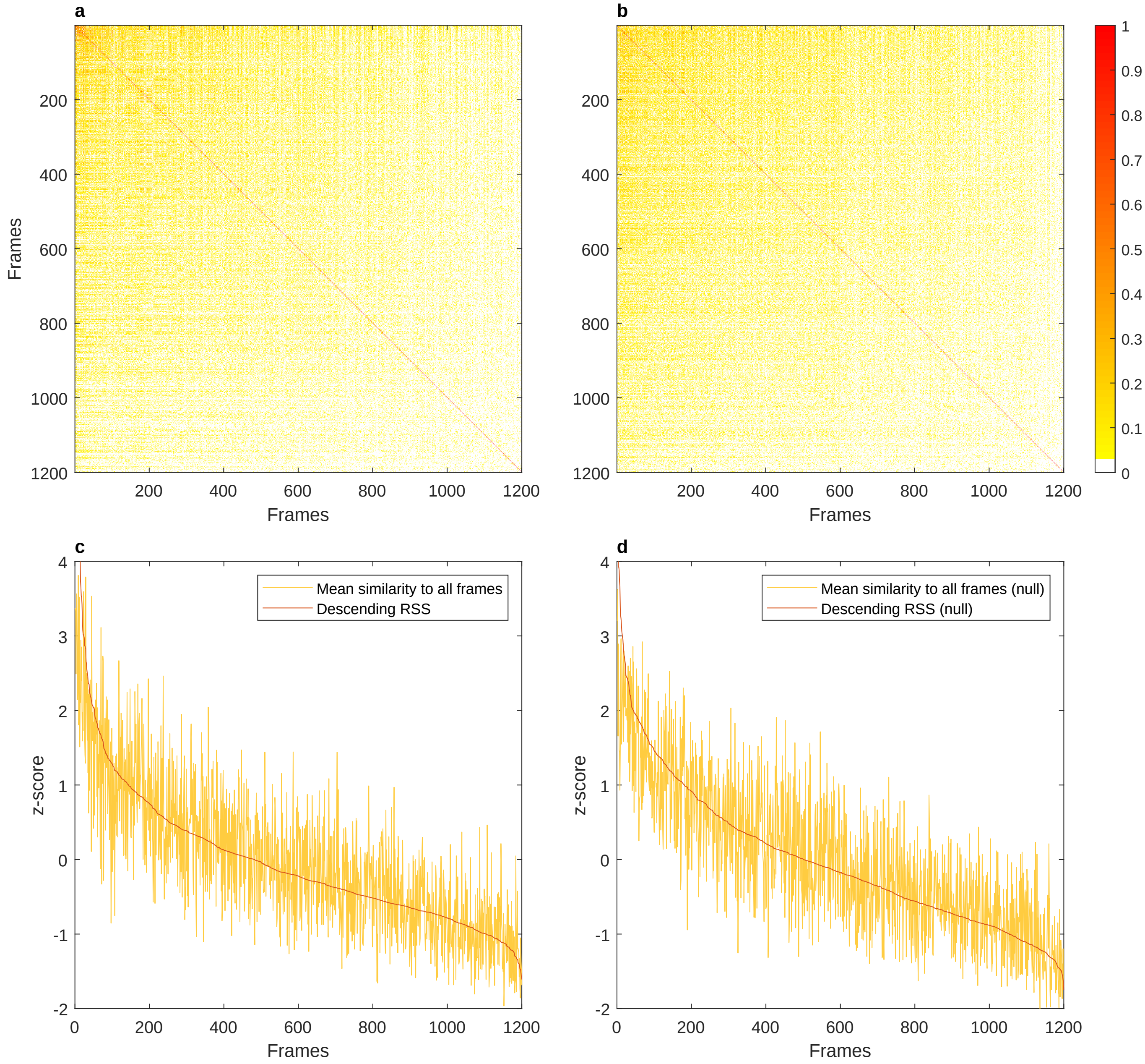

Supplementary Fig. 3. Relationship between RSS magnitude and average similarity to all frames. **a)** Correlation between all pairs of cofluctuation patterns for a single HCP subject (ID 101915). Frames are arranged in descending order of RSS magnitude so that high-RSS frames are in the top-left corner. This is akin to rearranging the rows and columns of the FC dynamics matrix used in sliding-window approaches to time-varying FC [50]. **b)** Corresponding null model results. **c)** Averaging the columns of the similarity matrix in panel **a** yields the mean similarity to all frames. The similarity decreases with the RSS, which is plotted in red. **d)** Corresponding null model results.

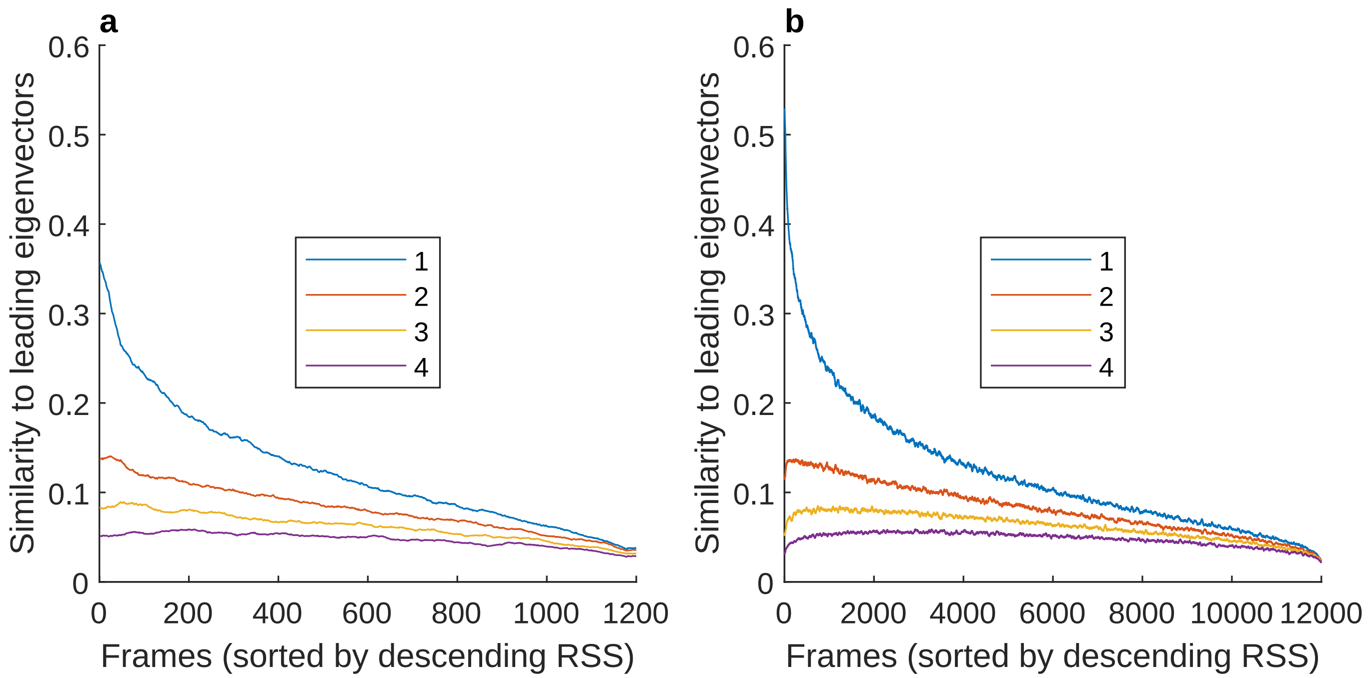

Supplementary Fig. 4. Frames corresponding to large RSS values exhibit high similarity to the leading nFC eigenvectors. This similarity increases with the number of frames, as shown by comparing the empirical and synthetic results. **a**) Empirical results using 1200 frames available in the HCP dataset, as in Fig. 5b in the main text. The similarity is measured as the Pearson correlation between the FC estimate from a single frame and each of the estimates from the the four leading nFC eigenvectors. **b**) The same analysis was repeated on 10 times longer synthetic data (i.e. 12 000 frames compared to previously analysed 1200 frames) generated for each HCP subject using their empirical nFC as input to the null model. The empirical nFC of a subject was set as the covariance matrix of a multivariate Gaussian process, which was sampled to obtain a longer synthetic scan for that subject.

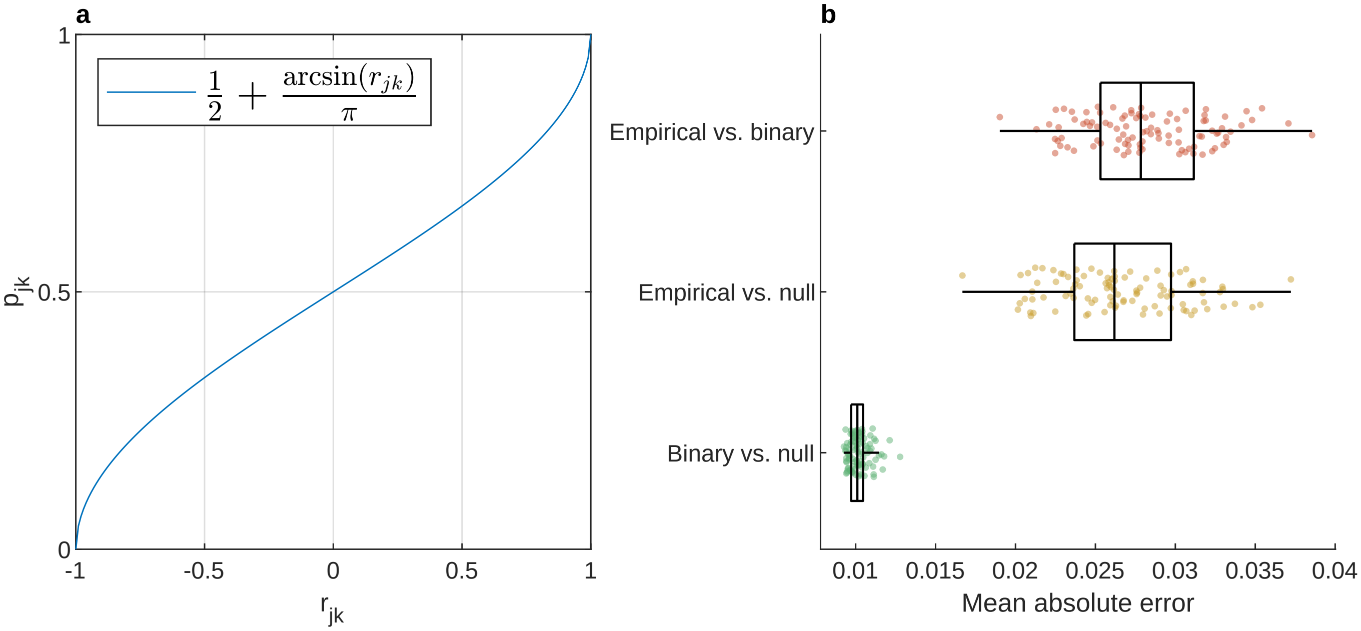

Supplementary Fig. 5. Binary edge time series properties. **a)** Under the static Gaussian null model, the binary edge time series can be modelled as Bernoulli(p_{jk}) random variables, where the success probability p_{jk} is a function of the Pearson correlation coefficient r_{jk}. The plot confirms and extends the intuition that $p_{jk} = 0$ for anticorrelated variables (parcels) and $p_{jk} = 1$ for perfectly correlated ones, crossing $p_{jk} = 0.5$ for uncorrelated variables. **b)** Averaging the binary edge time series over time yields a good approximation of the empirical nFC matrix computed on the HCP dataset, as measured by the mean absolute error (top box-whiskers plot, red dots). A similar error is obtained when comparing the nFC with the null model prediction produced using the function plotted in panel **a** (middle box-whiskers plot, yellow dots). Comparing the null prediction to the average binary time series results in an even lower mean error, validating the analytic derivation (bottom box-whiskers plot, green dots). Note that the empirical nFC must be rescaled to the $[0, 1]$ interval to allow for a direct comparison with the average binary edge time series and the analytic null probability. Each point corresponds to one of 100 unrelated subjects from the HCP, with boxes indicating the quartiles and whiskers length specified as 1.5 times the interquartile range.

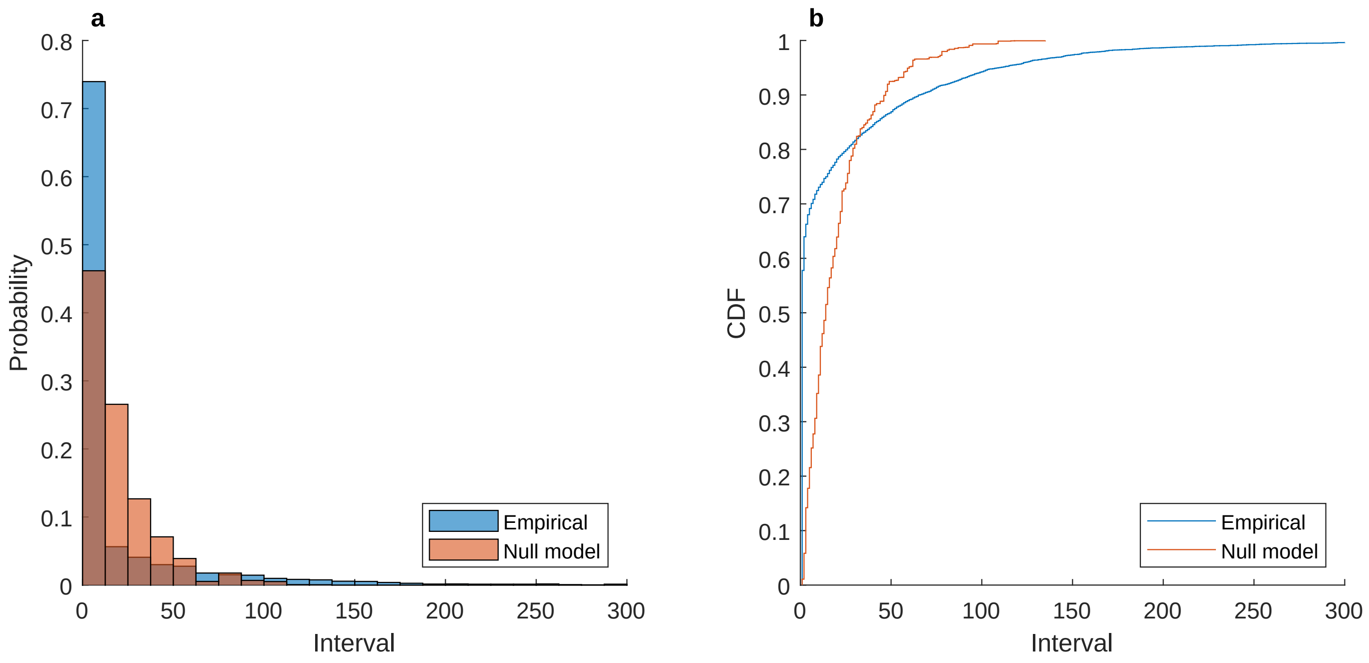

Supplementary Fig. 6. Distribution of time intervals between high-amplitude cofluctuations, computed across 100 unrelated HCP participants. The relative probability and cumulative distribution function (CDF) are reported in panels **a** and **b**, respectively. The root-sum-of-squares (RSS) of the edge time series was computed at each time step and the intervals between large realisations (those above the 95th percentile) were computed. The null model only captures the observed spatial correlations and not the temporal ones; therefore, it cannot reproduce the empirical distribution of the intervals.
