# Supplementary material for: A mathematical perspective on edge-centric brain functional connectivity
Source: Nat Commun. 2022 May 16;13:2693. doi: 10.1038/s41467-022-29775-7 (PMC9110367; doi:10.1038/s41467-022-29775-7)
Supplement: Supplementary file 2 — Reporting Summary [file 41467_2022_29775_MOESM2_ESM.pdf]

nature portfolio

Corresponding author(s): Leonardo Novelli

Last updated by author(s): Feb 24, 2022

Reporting Summary

Nature Portfolio wishes to improve the reproducibility of the work that we publish. This form provides structure for consistency and transparency in reporting. For further information on Nature Portfolio policies, see our Editorial Policies and the Editorial Policy Checklist.

Statistics

For all statistical analyses, confirm that the following items are present in the figure legend, table legend, main text, or Methods section.

n/a	Confirmed	
☐	☒	The exact sample size (n) for each experimental group/condition, given as a discrete number and unit of measurement
☐	☒	A statement on whether measurements were taken from distinct samples or whether the same sample was measured repeatedly
☐	☒	The statistical test(s) used AND whether they are one- or two-sided<br>*Only common tests should be described solely by name; describe more complex techniques in the Methods section.*
☒	☐	A description of all covariates tested
☐	☒	A description of any assumptions or corrections, such as tests of normality and adjustment for multiple comparisons
☐	☒	A full description of the statistical parameters including central tendency (e.g. means) or other basic estimates (e.g. regression coefficient) AND variation (e.g. standard deviation) or associated estimates of uncertainty (e.g. confidence intervals)
☐	☒	For null hypothesis testing, the test statistic (e.g. F, t, r) with confidence intervals, effect sizes, degrees of freedom and P value noted<br>*Give P values as exact values whenever suitable.*
☒	☐	For Bayesian analysis, information on the choice of priors and Markov chain Monte Carlo settings
☒	☐	For hierarchical and complex designs, identification of the appropriate level for tests and full reporting of outcomes
☐	☒	Estimates of effect sizes (e.g. Cohen's d, Pearson's r), indicating how they were calculated

Our web collection on statistics for biologists contains articles on many of the points above.

Software and code

Policy information about availability of computer code

Data collection	No software was used for data collection as the Human Connectome Project dataset is publicly available and was not collected as part of this study.
Data analysis	Data analysis was performed with MATLAB (MathWorks, Inc., version 2020b) and the code is made freely available on Github: https://github.com/LNov/eFC. Standard built-in functions are used to compute the singular value decomposition and to run the k-means algorithm. The HCP preprocessing pipelines code is also available on GitHub: https://github.com/Washington-University/HCPpipelines

For manuscripts utilizing custom algorithms or software that are central to the research but not yet described in published literature, software must be made available to editors and reviewers. We strongly encourage code deposition in a community repository (e.g. GitHub). See the Nature Portfolio guidelines for submitting code & software for further information.

Data

Policy information about availability of data

All manuscripts must include a data availability statement. This statement should provide the following information, where applicable:

- Accession codes, unique identifiers, or web links for publicly available datasets
- A description of any restrictions on data availability
- For clinical datasets or third party data, please ensure that the statement adheres to our policy

The preprocessed imaging data from the Human Connectome Project is publicly available and can be accessed after signing a data use agreement at https://db.humanconnectome.org.

Field-specific reporting

Please select the one below that is the best fit for your research. If you are not sure, read the appropriate sections before making your selection.

☒ Life sciences ☐ Behavioural & social sciences ☐ Ecological, evolutionary & environmental sciences

For a reference copy of the document with all sections, see nature.com/documents/nr-reporting-summary-flat.pdf

Life sciences study design

All studies must disclose on these points even when the disclosure is negative.

Sample size	This study used openly-available and independently-acquired resting-state fMRI data from the Human Connectome Project (HCP) S1200 release. In particular, the "100 unrelated subjects" dataset was used: a subset of 100 unrelated adult participants (among a larger group of twins recruited) which were pre-selected by the HCP coordinators (54% female; mean age =29.11 +- 3.67 years; age range, 22-36 years). This choice is based on previously published studies that used the same Human Connectome Project dataset (Esfahlani et al. 2020, Faskowitz et al. 2020, see references in the manuscript) and allows for a direct comparison of the results.
Data exclusions	No data was excluded from the study.
Replication	n/a
Randomization	Subjects were not allocated to groups and no randomisation was performed.
Blinding	Blinding was not applicable as subjects were not allocated to groups.

Reporting for specific materials, systems and methods

We require information from authors about some types of materials, experimental systems and methods used in many studies. Here, indicate whether each material, system or method listed is relevant to your study. If you are not sure if a list item applies to your research, read the appropriate section before selecting a response.

Materials & experimental systems

n/a	Involved in the study	
☒	☐	Antibodies
☒	☐	Eukaryotic cell lines
☒	☐	Palaeontology and archaeology
☒	☐	Animals and other organisms
☐	☒	Human research participants
☒	☐	Clinical data
☒	☐	Dual use research of concern

Methods

n/a	Involved in the study	
☒	☐	ChIP-seq
☒	☐	Flow cytometry
☐	☒	MRI-based neuroimaging

Human research participants

Policy information about studies involving human research participants

Population characteristics	This study used openly-available and independently-acquired resting-state fMRI data from the Human Connectome Project (HCP) S1200 release. In particular, the "100 unrelated subjects" dataset was used: a subset of 100 unrelated adult participants (among a larger group of twins recruited) which were pre-selected by the HCP coordinators (54% female; mean age =29.11 +- 3.67 years; age range, 22-36 years).
Recruitment	HCP subjects were recruited from the Missouri Department of Health and Senior Services Bureau of Vital Records. The authors of the present study did not collect any of the primary imaging data nor did they conduct recruitment of subjects.
Ethics oversight	HCP was approved by the Washington University Institutional Review Board.

Note that full information on the approval of the study protocol must also be provided in the manuscript.

Magnetic resonance imaging

Experimental design

Design type	Resting-state acquisition with eyes open and relaxed fixation on a projected bright cross-hair on a dark background (presented in a darkened room).

Design specifications | Resting-state fMRI data was acquired in four runs of 15 minutes over a 2-day period. Only the first 15-minute run for each participant was used in this study.

Behavioral performance measures | No behavioral measures were used in this study.

Acquisition

Imaging type(s) | Resting state BOLD

Field strength | 3T

Sequence & imaging parameters | All subjects were scanned on a customized Siemens 3T "Connectome Skyra" with a 32-channel head coil, housed at Washington University in St. Louis. Resting state images were collected with the following parameters: gradient-echo EPI sequence, run duration = 14:33 min, TR = 720 ms, TE = 33.1 ms, flip angle = 52°, FOV = 208x180 mm (RO x PE), matrix = 104x90 (RO x PE), slice thickness = 2 mm, 2-mm isotropic voxel resolution, multi-band factor = 8, echo spacing = 0.58 ms, BW = 2290 Hz/Px).

Area of acquisition | Whole brain

Diffusion MRI ☐ Used ☒ Not used

Preprocessing

Preprocessing software | We used the preprocessed and cleaned imaging data made available as part of the HCP and did not conduct further preprocessing, except for global signal regression where indicated. The preprocessing scripts are openly available on Github (https://github.com/Washington-University/HCPpipelines). The HCP S1200 release reference manual (https://humanconnectome.org/study/hcp-young-adult/document/1200-subjects-data-release/) indicates that functional images in the HCP dataset were minimally pre-processed according to the pipeline described in Glasser et al. (2013). The data was corrected for gradient distortion, susceptibility distortion and motion and then aligned to a corresponding T1-weighted image with one spline interpolation step.

Normalization | As part of the HCP pipelines referenced above, the volume was further corrected for intensity bias and normalised to a mean of 10000.

Normalization template | As part of the HCP pipelines referenced above, the volume was projected to the 32k_fs_LR mesh (excluding outliers), and aligned to a common space using a multi-modal surface registration.

Noise and artifact removal | As part of the HCP pipelines referenced above, the preprocessed rsfMRI data was cleaned of structured noise through a process that pairs independent component analysis (MELODIC) with FIX to automatically remove non-neural spatiotemporal components (trained on 25 hand-labeled HCP subjects). The FIX approach and initial results of classification accuracy are detailed in Salimi-Khorshidi et al. (2014), and the effects of the ICA + FIX cleanup (and optimal methods to remove the artefactual components from the data) are evaluated in detail in Griffanti et al. (2014). The cleaning pipeline is described more comprehensively in the HCP S1200 release reference manual (https://humanconnectome.org/study/hcp-young-adult/document/1200-subjects-data-release/) and the preprocessing and cleaning scripts are openly available on Github (https://github.com/Washington-University/HCPpipelines).

Volume censoring | No further volume censoring was applied.

Statistical modeling & inference

Model type and settings | Independent and identically-distributed (i.i.d.) multivariate Gaussian random variables are used as a null model for the parcellated BOLD signal.

Effect(s) tested | The empirical RSS distribution is tested against the null distribution using a two-sided Kolmogorov-Smirnov test (nonparametric).

Specify type of analysis: ☒ Whole brain ☐ ROI-based ☐ Both

Statistic type for inference (See Eklund et al. 2016) | Kolmogorov–Smirnov statistic

Correction | FDR (critical significance threshold set to 0.05)

Models & analysis

n/a	Involved in the study
☐	☒ Functional and/or effective connectivity
☐	☒ Graph analysis
☐	☒ Multivariate modeling or predictive analysis

Functional and/or effective connectivity | Functional connectivity is estimated via Pearson correlation and eFC (defined in the Methods section and

Functional and/or effective connectivity	similar to Pearson correlation).
Graph analysis	Community detection is performed applying the k-means clustering algorithm to the eFC matrix. This could be seen as a type of higher-order graph analysis.
Multivariate modeling and predictive analysis	i.i.d. multivariate Gaussian random variables are used as a null model for the parcellated BOLD signal.
